# The Abuse of Athletic Games

**Published:** 1869-01

**Authors:** 


### The Abuse of Athletic Games.

The same spirit of misdirected emulation which is the pest of our school system, has invaded a most wholesome public movement towards the cultivation of manly sports. The starved muscles and hypertrophied brains of our school-children seemed about to experience some restoration of the healthy balance of the faculties by a resort to exercise and out of door amusements. But, unfortunately, the same over-stimulated desire to excel, which converts the one into a precocious mental dwarf, drives the other to an expansion of bone and muscle beyond its natural growth. Pedestrians, oarsmen, base-ball players and match skaters, run to as great extremes and with as bad effects, as the phenomena of the schools who scan a Greek Idyl, and ignore the existence of Burns and Goldsmith; or measure the orbit of Uranus, but miscount the fractions in the simplest calculations of every-day life. He who walks, must walk for a wager; he who rows, must train for a prize; he who plays ball, must dislocate his fingers or break his nose in the false ambition to outdo his antagonist, while girls over-strain their slender ankles, and possibly derange the pelvic organs by emulous rivalry beyond their strength, either on skates with the mercury at zero, or in the crowded German, in a temperature of eighty-five degrees.

Neither time nor courage avail us to assail that compound evil of our school system alike defended by committees, teachers, parents and scholars, but secretly deprecated by all.

But as mentors of the public health we would say a word on the abuse of athletic games.

It has been a subject of general congratulation of late years, that somewhat of English customs in out of door sports was beginning to manifest itself among us, to the obvious physical advantage of our youth.

It was noticeable that the present generation of town-bred boys and girls, was not only rosier, stronger and manlier than the one before it, but even surpassed the average of country children in vigor. No one has been a warmer advocate of the use and pursuit of exercise than our own profession. We object only to its abuse.

The period of adolescence is the period of natural growth, but it is also easily overstrained and perverted. The war proved this; for many a young soldier, whose epiphyses were yet green and weakly soldered, broke down with joint diseases after protracted marches.

It cannot be a matter of doubt that like injury may follow excessive gymnastics, rowing or walking. Parts which are growing, are unfinished and weak.

If we do not give nature time to complete her work, but make demands on her, which only the mature man can fulfill, we surely defeat our object of promoting physical development. The child, left to itself, runs, plays, climbs, falls, with impunity, because it rests when it is tired, and stops when exhausted. So should the youth who seeks for muscular strength. But in the gymnasium, on the ball-ground, or in the wherry, he forgets fatigue in excitement, and he overdoes his muscles and his nervous power. The result is prostration, and not strength. He seeks to keep up his flagging powers by an absurd system of training, so called, where he is subjected to the caprice of certain physical sages, ignorant of physiology and of hygiene.

No college boy can rival the fisherman at the oar, permanently, because the latter has been in slow and gradual training all his life. Muscular growth, stiffening of bones, toughening of fibre and fascia must come slowly, in order to last. Only in the steady physical laborer can we find that harmonious development which combines strength, wind and endurance. We make a mistake if we expect to attain it in three months. For one part is then developed at the expense of another, and either the joints, the lungs, the heart, or the spinal system suffer in the unequal struggle.

We would by no means be classed with those who would restrain either sex from out of door pursuits. We would drive every boy and girl into the open air several hours a day. We would say, row, walk, swim, skate, and play every hardy game. But do so reasonably, and do not seek to make them the business of life, by a few weeks' pursuit. Make haste slowly. Give your limbs time to rest, and they will grow.

If you want to become an athlete, follow the trade of the fisherman, or the day-laborer, or the organ-grinder. If you have other aims in life, and mean to use your minds as well as your bodies, give time and cultivation to both, at due intervals. But do not expect that you can over-develop the one without dwarfing the other. Intellectual culture alone will make you a nervous, unbalanced, precocious man. Physical culture alone will make you as strong as the hod-carrier—and as dull.—*Editorial Boston Medical Journal.*

